# Directed Surface Immobilization of Coordination Cages for Host–Guest Studies

**DOI:** 10.1021/acs.langmuir.6c03872

**Published:** 2026-09-29

**Authors:** Samantha Muhring-Salamone, Lisa Schardt, Shota Hasegawa, Alexandre Walther, Björn Schmidt, Paul D. Ashby, Guido H. Clever, Axel Rosenhahn

**Affiliations:** † Analytical Chemistry − Biointerfaces, 9142 Ruhr University Bochum, Bochum 44780, Germany; ‡ Department of Chemistry and Chemical Biology, 14311 TU Dortmund University, Dortmund 44227, Germany; § Molecular Foundry, 1666 Lawrence Berkeley National Laboratory, Berkeley, California 94720, United States

## Abstract

Self-assembled coordination cages are bioinspired host–guest systems mimicking natural nanoconfinements and their functions as receptors and reaction compartments. While such systems are conventionally studied in solution or as bulk materials, the examination of surface-immobilized cages has recently emerged as an attractive approach. Here, we investigated the directed immobilization of bowl-shaped coordination cages [Pd_2_L_3_(MeCN)_2_]^4+^ on carboxy-terminated self-assembled monolayers. Spectroscopic ellipsometry and in situ flow experiments using SPR spectroscopy were used to characterize the immobilization of the coordination cages from the solution. ATR-FTIR confirmed the chemical identities of the surface-bound structures. Encapsulation of C_60_ in the coordination cages revealed faster surface absorption, consistent with an increase in binding affinity. Immobilized cages containing C_60_ guests were imaged by atomic force microscopy and revealed surface structures with dimensions consistent with those of the coordination cages, indicating successful capture on self-assembled monolayers.

## Graphical abstract

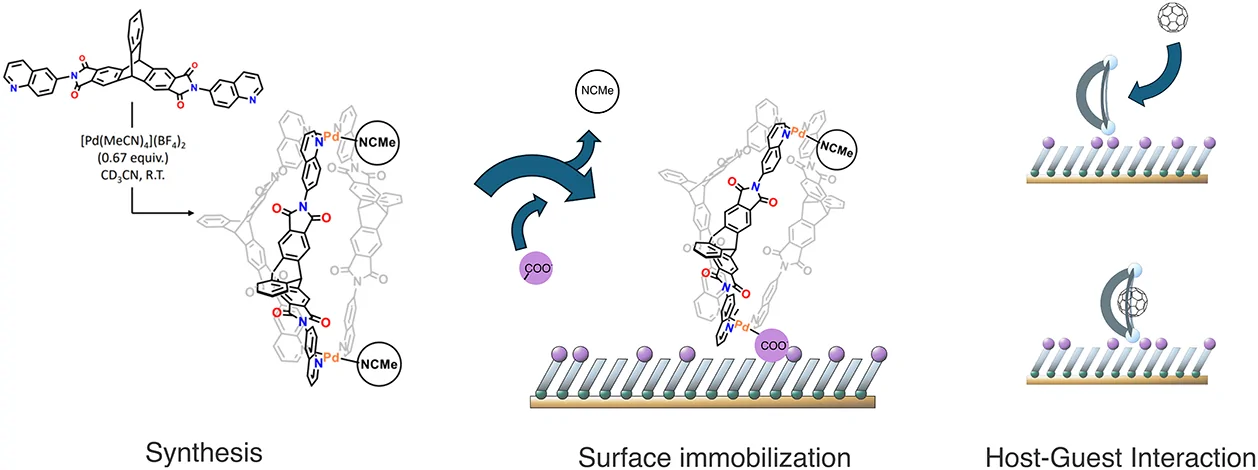

## Introduction

Supramolecular coordination cages are discrete, three-dimensional, self-assembled systems that feature carefully selected metal centers coordinated by bridging ligands.^1^ A distinction is made between homoleptic and heteroleptic coordination cages, based on whether all ligands are identical or different ligands coordinate to the metal centers.^2,3^ Many coordination cages are designed as host–guest systems to mimic natural processes such as selective recognition and functions such as enzymatic catalysis.^4^ Their three-dimensional structure creates a well-defined open cavity at the center of the cage, enabling guest uptake and release.^5–7^ Those self-assembled host–guest systems have primarily been studied in solution or as bulk materials, but the examination of surface-immobilized supramolecular coordination cages (SurSCCs) has recently emerged as a promising extension, allowing for single-molecule studies and enabling the direct investigation of their structural and functional properties at interfaces.^7,8–11^ One method to immobilize supramolecular coordination cages on surfaces was recently published by Menozzi et al., in which they synthetically equipped a cage with a long alkyl tether, which enabled surface immobilization via van der Waals interactions with the alkyl chains of a surface-bound self-assembled monolayer (SAM).^8^ Frank et al. deposited coordination cages on nanostructured Au surfaces and used Raman and SERS spectroscopy to demonstrate the ability to distinguish between the free ligand and the ligand embedded in the supramolecular structures. The results provided clear evidence for the integrity of surface-deposited coordination cages. Ryan et al. demonstrated the absorption of coordination cages to activated alumina from solution, revealing the integrity of the supramolecular structures after immobilization while still retaining their ability to bind and release guest molecules.^10^

A frequently used guest in supramolecular coordination cages is the spherical Buckminsterfullerene C_60_. The special curved molecule with a fully π-conjugated surface inspired many scientists to investigate its properties, its binding properties as guest, derivatizations, and applications.^12,13^ Its unique electronic properties as an electron acceptor and an electron transport molecule led to several applications in material science, photovoltaics, and organic electronics.^14,15^ The versatility of C_60_ has led to interest in its controlled derivatization, preparation of composite materials, surface deposition, and purification. One motivation for this research was to identify alternatives to the tedious and costly selective purification of mixtures of fullerenes and their derivatives by chromatography.^14,16^ Therefore, a wide variety of fullerene-encapsulating supramolecular coordination cages have been constructed over the past decades.^17,18^ For fullerene guest binding, the host requires a relatively large cavity^14^ which can make it susceptible to the nonspecific binding of other guests.^17,18^ In order to achieve selective recognition of fullerenes, extensive π–π interactions, supported by CH−π interactions and solvophobic effects, are often exploited to maximize the selectivity.^17,19^ One of the first examples of a covalent molecular receptor for fullerene was described in 1992 by Ringsdorf^20^ and Wennerström,^21^ who complexed a fullerene with aza-crown compounds and γ-cyclodextrins. Self-assembled coordination cages capable of binding fullerenes have been reported by several groups, including Nitschke as well as Yoshizawa and coworkers, the latter demonstrating polyaromatic capsules capable of encapsulating one or even two fullerene guests.^22,23^ Hasegawa et al. designed Pd_2_L_4_ lantern-shaped cages with triptycene-based ligands and pyridine donors coordinated to the Pd­(II) cations.^24^ The triptycene backbones offer a π-surface area, giving the ligand the perfect shape complementarity to the globular C_60_. The cage revealed its ability to stabilize radical intermediates, such as C_60_^•–^, an important charge-carrier state in light harvesting and electronic materials based on fullerenes.^16,24^ Chen et al. designed similar ligands based on a curved dibenzo-2.2.2-bicyclo-octane backbone, reminiscent of the triptycene backbone but containing additional sideways-pointing methyl substituents.^6^ They compared ligand derivatives containing pyridine donors with ligands in which the pyridines were replaced with quinolines. Surprisingly, those quinoline-based ligands led to open Pd_2_L_3_(CH_3_CN)_2_ bowl-like structures, where both metals are only bridged by three instead of four ligands, with a solvent molecule coordinating to the fourth coordination site of both Pd cations. This structure allowed the monofunctionalization of fullerene guests with bulky substituents on the part of the fullerene surface not protected by the bowl, showing the host’s potential to steer fullerene reactivity.^15^ Furthermore, it has been shown that C_60_ encapsulation can improve its solubility in a wide range of solvents, thus enabling the use of more polar organic solvents and thereby allowing a wider range of reagents to be used in fullerene chemistry.^14^

Here, we introduce a method for the directed immobilization of a coordination cage on surfaces. Therefore, ligands were synthesized based on the triptycene-based ligands previously published by Hasegawa et al.^24^ by replacing the previously used pyridine groups with quinoline groups. A combination of the ligands with [Pd­(CH_3_CN)_4_]­(BF_4_)_2_ resulted in Pd_2_L_3_(CH_3_CN)_2_ SCCs with an open-bowl-like structure. Based on the previous observation that the loosely bound acetonitriles can be replaced by more strongly binding carboxylates,^15^ a carboxy-terminated self-assembled monolayer (SAM) was used to immobilize the coordination cages. We here show that indeed the exchange of the solvent molecules occupying the fourth coordination site of the Pd­(II) cations with the terminal carboxy groups of the SAMs results in the directed coordination of the bowl-shaped coordination cages to the surface. Using contact angle goniometry, spectroscopic ellipsometry, surface plasmon resonance spectroscopy (SPR), atomic force microscopy (AFM), and ATR-FTIR spectroscopy, the immobilization of the cages on the SAMs and the incorporation of C_60_ as its guest were analyzed.

## Materials and Methods

### Chemicals

11-Mercaptododecanoic acid (MDDA, Prochimia Surfaces, Poland), 16-Mercaptohexadecanoic acid (MHDA, Prochimia Surfaces, Poland), 1-Dodecanethiol (DDT, Prochimia Surfaces, Poland), ethanol (99%, VWR Chemicals, Radnor, USA), acetonitrile (Sigma-Aldrich, St. Louis, USA), and ultrapure water were purified with a Milli-Q system (Siemens Water Technology). For SPR measurements, glass substrates (1 cm × 1 cm, Schott, Germany) were precoated with 5 nm of titanium as an adhesion promoter and 60 nm of gold. For ATR-FTIR measurements, silicon wafers (Siegert Wafer, Germany) were precoated with 5 nm of titanium and 200 nm of gold.

### Bowl Synthesis

The ligand for the coordination cages was synthesized by condensing 2,3,6,7-triptycenecarboxylic dianhydride with 6-aminoquinoline. The ligand was stirred overnight with [Pd­(CH_3_CN)_4_]­(BF_4_)_2_ in a 3:2 ratio in acetonitrile at room temperature.

### Preparation of the Self-Assembled Monolayers on Gold

The anionic MDDA and MHDA and the uncharged DDT were allowed to self-assemble on gold and were subsequently used to immobilize the supramolecular bowl-shaped systems. The SAMs were dissolved in ethanol (2 mM); for the anionic SAMs, ammonia solution (3 vol%) was added. Mixed SAMs were prepared to adjust the number of terminating carboxyl groups on the surface of the SAMs. Therefore, DDT and the used anionic MDDA or MHDA thiols were mixed in different ratios. Gold substrates were annealed to create 100 nm grains with (111) facets parallel to the substrate surface. The gold substrates were irradiated with UV light for 1 h, then rinsed with p.a. ethanol, sonicated in p.a. ethanol for 3 min, and then rinsed again with p.a. ethanol. Depending on the used thiol, the substrates were incubated in the prepared solutions for 24 h (DDT) or 48 h (MDDA, MHDA, and mixed SAMs). Afterward, the substrates were rinsed with p.a. ethanol, sonicated for 3 min, and rinsed again with p.a. ethanol. The SAM substrates were dried under a nitrogen flow and stored under an argon atmosphere until they were used.

### Contact Angle Goniometry

The static water contact angles (WCA) of the SAMs were measured with a custom-built goniometer setup. Therefore, a water droplet was placed on the surface and recorded with a charge-coupled device (CCD) camera. A contour-tracing algorithm automatically distinguished the droplet from its mirror image and calculated the contact angle using Young’s equation. Data points were obtained from three individual measurements for each sample; the error represents the standard deviation.

### Spectroscopic Ellipsometry

The film thicknesses of the samples were measured by spectroscopic ellipsometry using an M-2000 V spectrometer (Woolam, Lincoln, USA). Measurements were acquired at three incidence angles (65°, 70°, and 75°). The thicknesses were modeled using a one-layer model with a wavelength depending refractive index described by the Cauchy model (A = 1.45, B = 0.1, C = 0). While for the self-assembled monolayers, the measurements provided accurate film thickness, the measurements on the bowls rather represent an estimate of the overall deposited materials than the true thickness and density of the assembled bowls. The reported values are the mean of at least three individual measurements. The error represents the standard deviations.

### Surface Plasmon Resonance Spectroscopy

Immobilization of the bowl-shaped coordination cages on self-assembled monolayers was analyzed by surface plasmon resonance (SPR) spectroscopy (SR7000DC, pump SR7500, autosampler SR7100, Reichert Technologies Life Science, USA). SPR chips (1 × 1 cm glass substrates, coated with 5 nm titanium as an adhesion promoter and 60 nm gold) were functionalized with self-assembled monolayers and installed in the spectrometer using immersion oil (n = 1.54). Acetonitrile was pumped through the system at 200 μL/min until a steady baseline was obtained. The flow rate was then reduced to 10 μL/min, and the sample solution (0.1–0.35 mM) was injected until equilibrium was reached. The system was subsequently washed with acetonitrile for at least 15 min at 10 μL/min until a steady baseline was again obtained. The difference in the signal before and after sample injection (ΔμRIU) indicates the amount of coordinated bowl on the surface. The kinetics in the SPR sensorgrams were evaluated by fitting the association phase with an exponential function. The fit parameters were optimized by minimizing the squared residuals between the experimental data and the fitted curves via nonlinear least squares (Microsoft Excel Solver). Association rate constants (*k*_a_) were derived from the optimized fit parameters.

### Attenuated Total Reflection-Fourier Transform Infrared Spectroscopy

Attenuated total reflection-Fourier transform infrared (ATR-FTIR) spectra were obtained using a nitrogen-purged Tensor 27 (Bruker, Billerica, USA) with a Diamond ATR accessory (100 scans, Golden Gate, Specac, UK) for pure samples and a Grazing Angle Accessory (300 scans, Ge ATR crystal, VariGATR, Harrick, USA) for the analysis of self-assembled monolayers and immobilized samples on silicon wafers. Spectra were recorded over a range of 4000–1050 cm^–1^ at a resolution of 4 cm^–1^ using a liquid nitrogen-cooled Mercury Cadmium Telluride (LN-MCT) detector.

### Atomic Force Microscopy

In situ AFM imaging was performed on an Asylum Cypher-ES AFM (Asylum Research, Oxford Instruments) equipped with a liquid cell, using tapping mode. The AFM probe consisted of a sharp silicon tip on a silicon nitride cantilever (BioLever Mini, BL-AC40TS, Olympus) with a spring constant of 0.09 N/m. A liquid-perfusion cantilever holder (Cypher VRS, Oxford) was used to reduce evaporation of the liquid during measurements and to enable ethanol injection into the AFM liquid cell via a syringe. Images were collected at line frequencies of 1–10 Hz with oscillation amplitudes of ∼3 nm.

## Results and Discussion

The ligand for the coordination cages was synthesized by condensing 2,3,6,7-triptycene carboxylic dianhydride with 6-aminoquinoline. Due to steric hindrance from the hydrogen atoms of the quinolines, an open, bowl-shaped [Pd_2_L_3_(MeCN)_2_]^4+^ coordination cage was formed, with two coordination sites occupied solely by the solvent.^15^ To immobilize the bowl-shaped coordination cage, self-assembled monolayers with a carboxylate termination were prepared. Because of the high affinity of the terminal carboxy group to Pd­(II), it is capable of displacing the coordinated solvent, thereby binding and tethering the coordination cage to the surface (Figure 1B).

**Figure 1:**
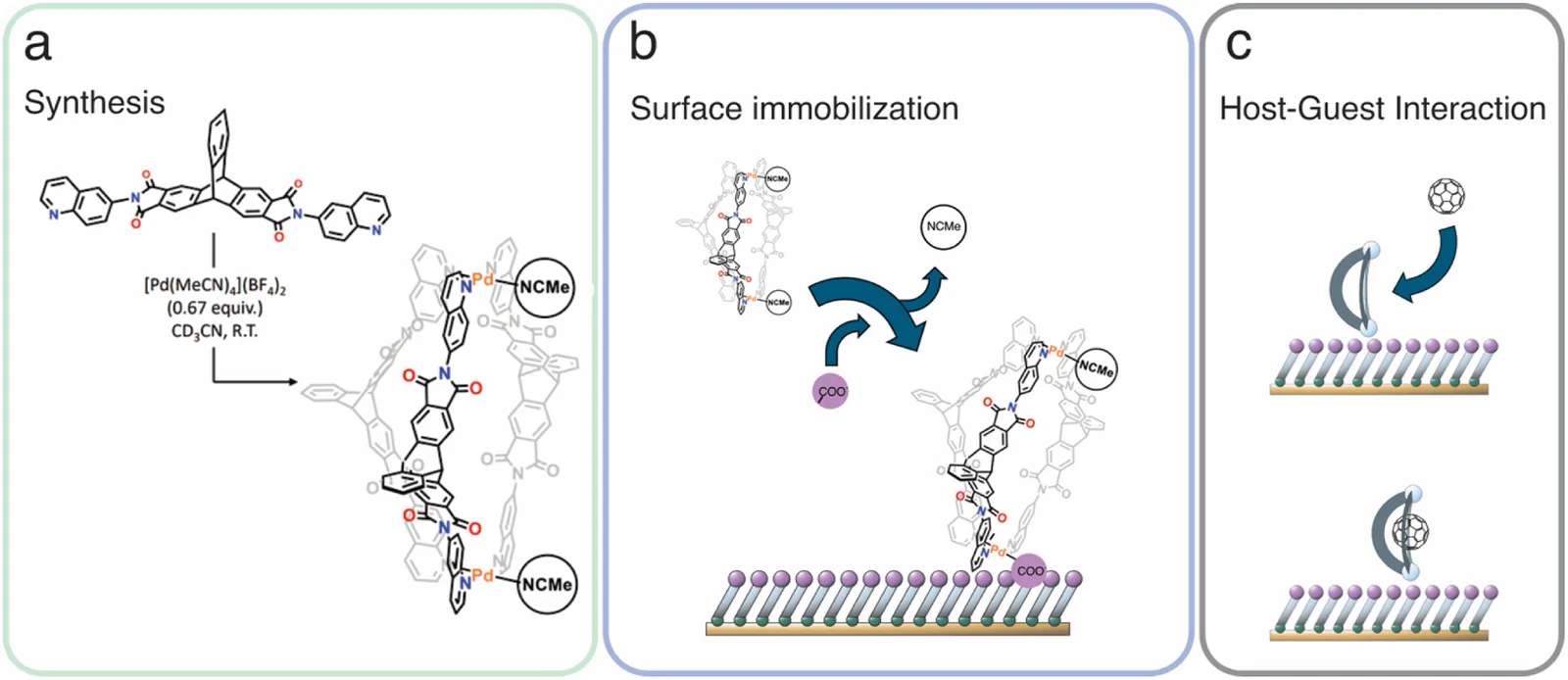
Schematic illustration of the directed immobilization of the bowl-shaped coordination cages. (a) Self-assembly of the bowl-shaped coordination cage. (b) Surface immobilization of the cages by exchanging the coordinated solvent with the carboxylic acid of the SAM surface. (c) Host–guest interaction of the cage with C_60_.

To investigate the immobilization of the bowl-shaped coordination cages on the prepared SAM surfaces, an in situ flow experiment was conducted using SPR spectrometry (Figure 2A). For comparison, solutions of the free ligand and the Pd salt were also examined to assess the binding behavior of the building blocks relative to that of the coordination cages. The change in SPR response following their injection was assessed, revealing the highest SPR response after injection of the bowl-shaped coordination cage with 1050 ΔμRIU, followed by the SPR response after Pd salt injection of 628 ΔμRIU, and with the lowest response after injection of the ligand of 159 ΔμRIU. The rergrams reveal differences in their progression and, therefore, in their association kinetics, indicating a higher affinity for the cage coordination due to its higher *k*_a_ value (Figure 2B). With spectroscopic ellipsometry, the thickness of the SAM surfaces was analyzed after incubation in the solutions of the coordination cage, the free ligand, and the Pd salt (Figure 2c). The smallest increase in thickness was exhibited after incubation in the ligand solution, with an increase of 0.11 nm; the incubation in Pd-salt solution led to an increase of 0.21 nm; and the highest increase in thickness was observed after incubation in the solution of the bowl-shaped coordination cage, with 0.64 nm. With water contact angle (WCA) goniometry, the change in wettability after incubation in the different absorbent solutions was analyzed (Figure 2e), revealing an increase in hydrophobicity for all samples after incubation. MDDA exhibited a WCA of 28°, which increased after incubation in the ligand solution to 37°, after Pd-salt solution to 42°, and after incubation in the bowl solution the WCA increased to 61°.

**Figure 2:**
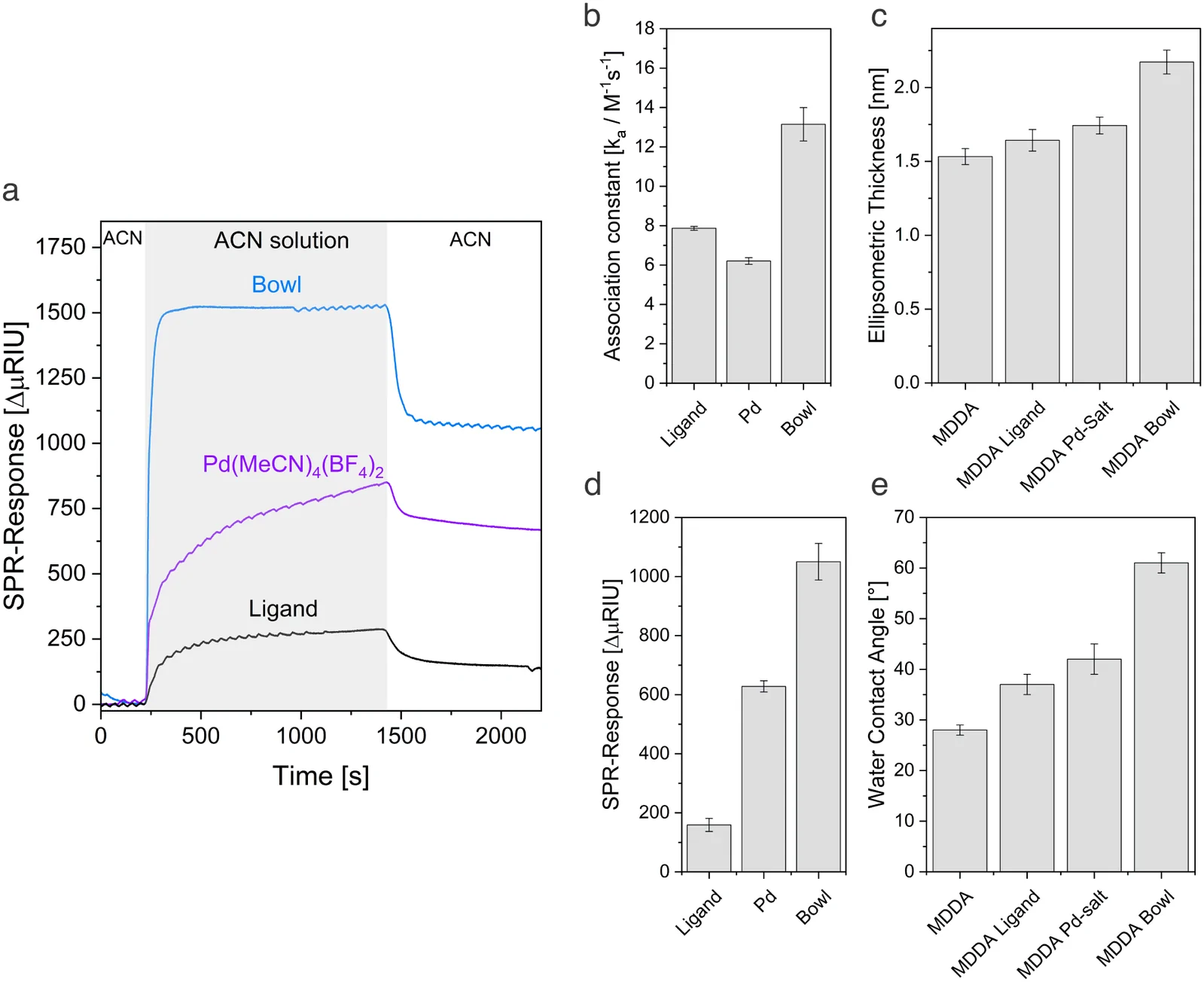
SPR analysis of the affinity of bowls, ligand, and palladium complex to surface-anchored carboxylate groups. (a) Representative SPR-sensorgram of the injection of 0.35 mM bowl solution, 1 mM ligand solution, and 0.2 mM Pd­(MeCN)_4_(BF_4_)_2_ solution in acetonitrile on an MDDA-functionalized SPR chip. Bowl solutions and solutions of their building blocks were injected for 20 min at a flow rate of 10 μL/min. (b) Association rate (*k*_a_) obtained from fitting the immobilization phase of the SPR sensorgrams. (c) Thickness of the SAM surface before and after incubation in solutions of the different adsorbents. Values are the mean of at least three individual measurements. The error bars represent the standard deviation. (d) SPR response of MDDA after injection of 0.35 mM bowl solution, 1 mM ligand solution, and 0.2 mM Pd­(MeCN)_4_(BF_4_)_2_ solution in acetonitrile. Samples were injected for 20 min with a flow rate of 10 μL/min. Values were obtained after dissociation at *t* = 2000 s. Values are the means of at least three individual measurements. The error bars represent the standard deviation. (e) Water contact angle of MDDA before and after incubation in the solutions of the different adsorbents. Values are the means of at least three individual measurements. The error bars represent the standard deviation.

ATR-FTIR analysis was used to investigate the adsorption of the bowl-shaped coordination cage onto the SAM surface (Figure 3). Therefore, IR spectra of the ligand and the bowl were measured, both as powders and surface-immobilized on gold-coated silicon wafers functionalized with MDDA, to allow comparison and identify changes induced by surface immobilization. Comparing the dry bowl and dry ligand IR spectra revealed very similar vibrational modes. Both spectra showed a peak around 1716 cm^–1^ and around 1773 cm^–1^ (Figure 3a), assigned to the symmetric and antisymmetric vibration of the C=O bond of the imide function (O=C–N–C=O).^25–27^ Both peaks broadened upon palladium coordination in the bowl system, and the peak at 1773 cm^–1^ shifted to 1776 cm^–1^ upon Pd coordination. The ligand spectrum showed a peak at 1504 cm^–1^ (Figure 3b), assigned to aromatic C=C stretching vibrations,^28^ which shifted to 1511 cm^–1^ for the bowl system, likely due to coordination of the quinoline moieties to the palladium metal center. This is consistent with previous reports of quinoline exhibiting shifts of around 8–11 cm^–1^ after coordination to M­(II) centers.^28^ The peak at 1465 cm^–1^ shifts to 1469 cm^–1^ for the coordinated cage and can be assigned to the aromatic ring vibration of the quinoline (Figure 3c). At 1362 cm^–1^ and 1380 cm^–1^ (Figure 3d), the bowl spectrum showed two peaks, whereas the ligand only shows one broad peak with its maximum at 1362 cm^–1^ and a shoulder at around 1375 cm^–1^. Those peaks can also be assigned to the deformation vibration of the aromatic ring structures. Literature has previously reported that coordination of a heteroatomic compound to an M­(II) metal center induces a shift of this peak by about 6–9 cm^–1^ to higher wavenumbers.^28^

**Figure 3:**
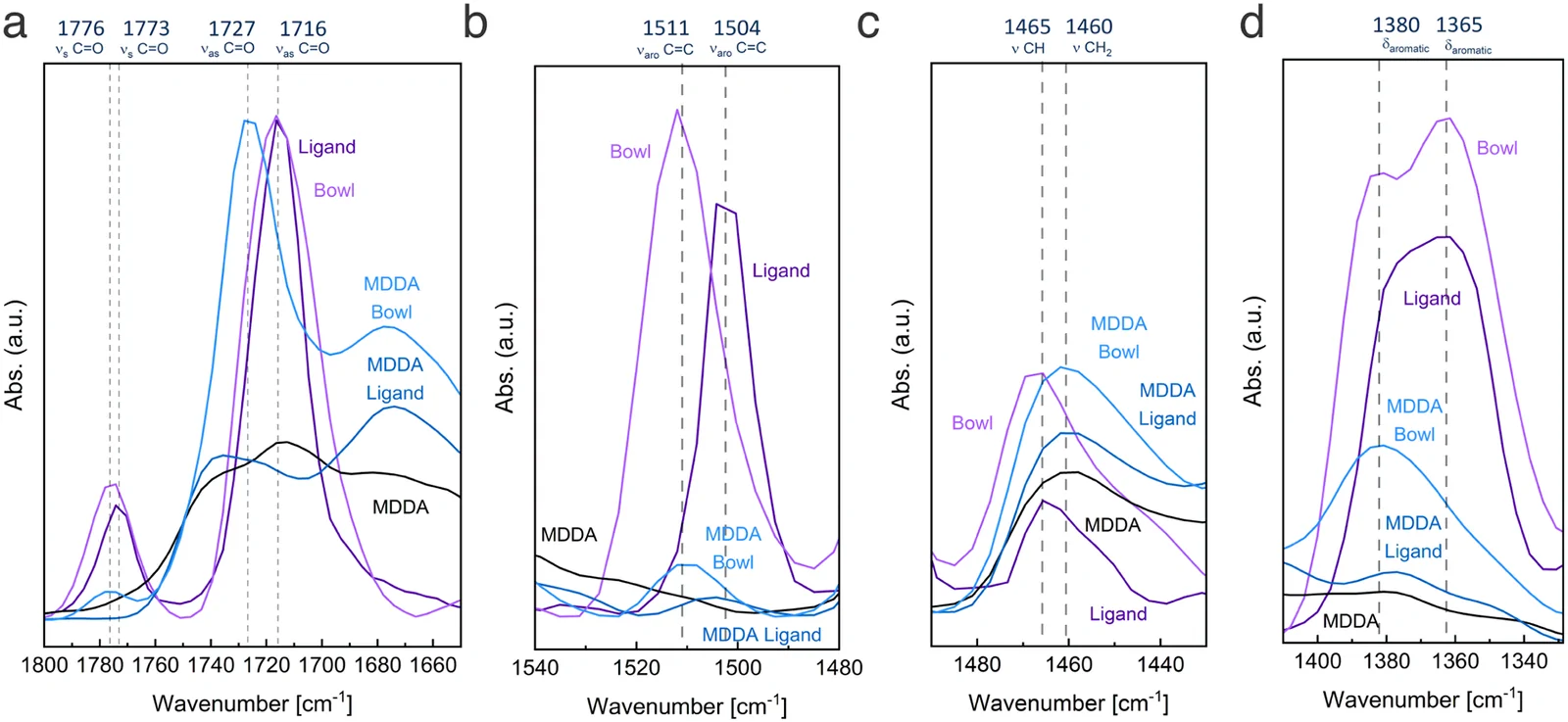
ATR-FTIR spectra of the bowl-shaped coordination cage and its ligand, the SAM surface MDDA, and the surface-absorbed coordination cage and ligand. Selected spectral regions are shown as insets: (a) 1800 to 1650 cm^–1^, (b) 1540 to 1480 cm^–1^, (c) 1490 to 1430 cm^–1^, (d) 1420 to 1330 cm^–1^.

After incubation of the SAM in the ligand solution, the peak assigned to the C=O vibration of the terminal carboxy group shifted from 1712 cm^–1^ (MDDA) to 1740 cm^–1^ (MDDA ligand) (Figure 3a), indicating a disturbance in the hydrogen bonding of the structured SAM surface.^29^ MDDA bowl shows a peak at 1776 cm^–1^ and at 1727 cm^–1^, which can be assigned to the symmetric and antisymmetric vibration of the C=O of the imide function of the coordination cage.^25^ The peak at 1727 cm^–1^ was seen at 1716 cm^–1^ for the nonimmobilized ligand and bowl, revealing a peak shift of 8–12 cm^–1^ to higher wavenumbers, indicating a change in the imide environment. The peak was shown before in the spectrum of the coordination cage itself and is not visible for the MDDA ligand. Additionally, after immobilization, a peak appears at 1673 cm^–1^ for MDDA bowl and MDDA ligand, most likely also attributed to a modified imide carbonyl environment arising from surface immobilization.

As seen previously for the nonimmobilized ligand and bowl, the MDDA ligand and MDDA bowl show peaks at 1504 cm^–1^ and 1511 cm^–1^, assigned to aromatic C=C stretching vibrations.^28^ (Figure 3b) The peak at 1460 cm^–1^, for MDDA bowl and MDDA ligand, can be assigned to the CH_2_ scissoring band of MDDA^29^ (Figure 3c). The peak at 1465 cm^–1^, assigned to the aromatic ring vibration,^28^ is not visible after surface immobilization of the bowl and the ligand. At 1380 cm^–1^ and 1375 cm^–1^ (Figure 3d), the spectrum of the MDDA bowl and MDDA ligand shows a peak, which can be assigned to the deformation vibration of the aromatic ring but also to the carboxylate functions on the surface; as they show the same shift in signal as observed for bowl and ligand, the peaks are more likely to be assigned to vibrations of the aromatic rings.

An approach to reduce the number of binding sites and thus the density of coordination cages on the surface was to produce mixed SAMs consisting of carboxyl-terminated MDDA or MHDA SAMs diluted by DDT. The DDT is purely aliphatic and methyl-terminated and thus unable to coordinate to the bowl-shaped coordination cages (Figure 4). In correlation with the content of the carboxy-terminated thiols in the mixed SAM, the wettability of the SAM increased, leading to an almost linear decrease in the water contact angle (WCA) from DDT of 103° to 31° for pure MDDA and 13° for pure MHDA (Figure 4a). Similar linear decreases in WCA were in the past observed for mixed SAM systems when the surface mixture changed linearly with the solution mixture ratio.^30^

**Figure 4:**
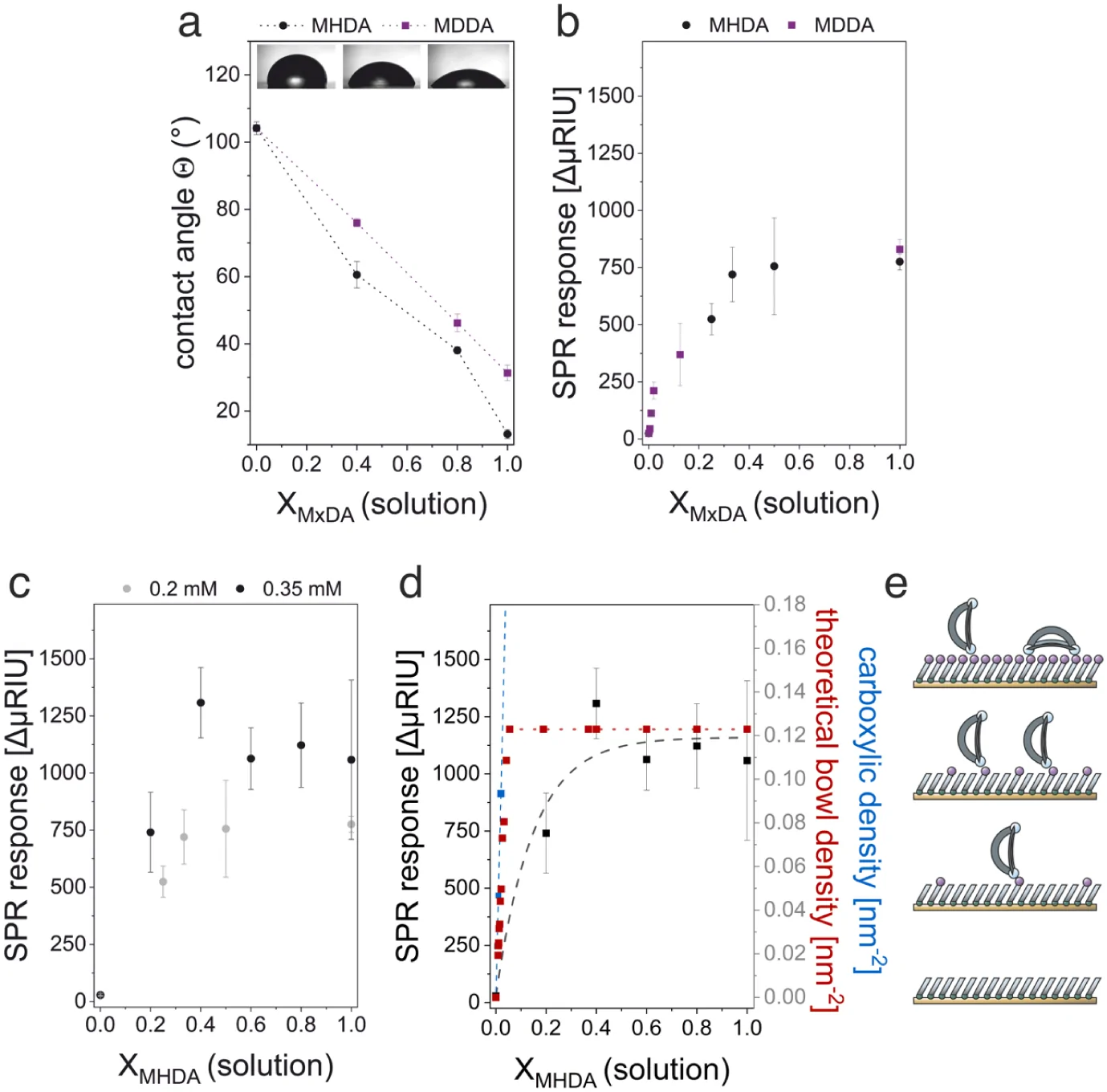
Variation of the carboxyl density in the self-assembled monolayers to control the SurSSCs density. (a) Water contact angle of the mixed SAMs with variation in the content of carboxylic acid in the prepared solution, where 0 is pure DDT and 1 is the pure carboxyl-terminated (MDDA squares and MHDA circles) SAM. (b) SPR response after injection of 0.2 mM bowl solution on different carboxylic SAMs: MHDA (violet) and MDDA (blue). SAMs with various molar ratios of DDT and carboxylic SAM (X_MxDA_) were used, where 0 is pure DDT and 1 is pure carboxylic SAM. Data points were obtained from three individual measurements per sample; the error represents the standard deviation. (c) SPR responses of mixed MHDA SAMs after injection of different concentrations of bowl solution: 0.2 mM (violet) and 0.35 mM (black). (d) Left *y*-axis: SPR response of mixed MHDA SAMs after injection of 0.35 mM bowl solution with different molar ratios of MHDA to DDT. The exponential fit of the data is plotted as a broken black line. Data points reflect the average of at least three individual measurements for each sample; the error represents its standard deviation. Right *y*-axis: carboxylic density of a SAM surface calculated by the packing density of the surface in blue and the theoretical bowl density bound to the surface in red. (e) Schematic illustration of mixed SAMs and the possible geometric arrangement of the bowl system with increasing carboxylic content on the surface.

As MHDA possesses a longer alkyl chain (HS-(CH_2_)_15_-COOH) compared to DDT (SH-(CH_2_)_11_-CH_3_) and MDDA (HS-(CH_2_)_11_-COOH), the carboxylic function of MHDA may stick out in such a mixed SAM surface. To investigate whether the longer chain length of the anionic SAM influences the coordination of the bowls to the surface, mixed SAMs with different ratios of MHDA/MDDA and DDT were prepared. The SPR response of the MDDA-based mixed SAMs was compared with the SPR response of the MHDA-based mixed SAMs after injection of 0.2 mM bowl solution (Figure 4b). The curves for both mixed SAM species initially show a steep rise, which then became increasingly flat and reached saturation at a carboxy-terminated SAM content of approximately 0.5 nm^–2^. The final SPR response in saturation was similar for both SAMs with 776ΔμRIU for MHDA and 830 ΔμRIU for MDDA for both pure carboxy-terminated SAMs.

To investigate the influence of the concentration of the injected bowl-shaped coordination cages on the absorption on mixed SAM surfaces, the SPR response was analyzed after injection of a 0.2 mM bowl solution and compared with that after injection of a 0.35 mM solution (Figure 4c). The injection of the solution with higher bowl concentration showed a higher maximum response of about 1100 ΔμRIU, which aligns with the SPR response of MDDA shown before (Figure 2a) of 1050 ΔμRIU. This maximum response occurred earlier than that after injection of the 0.2 mM bowl solution, which reached about 760 ΔμRIU.

Based on the hexagonal packing arrangement of the SAMs, the distance between neighboring terminal carboxy groups can be estimated from the literature to be ≈0.5 nm, as determined for alkanethiol SAMs.^31^ By assuming a similar assembly rate and packing density of MDDA, MHDA, and DDT, a theoretical density of the carboxylic groups on the surface of the mixed SAMs was calculated and plotted against the carboxylic content in the assembly solutions (X_MHDA_) (Figure 4d). A theoretical bowl density was calculated using the maximum geometrically possible distance for the carboxy functions on the SAM surface, along with the approximate dimensions of the bowl-shaped coordination systems. This approach shall reflect the spatial limitations set by the spatial demand of the coordination cages and the estimated coverage of the coordination systems on top of the mixed SAM. Up to a coverage of 0.12 nm^–2^, the theoretical maximum density of the coordination cages is limited by the number of available carboxylate groups in the mixed SAMs. For higher carboxylate densities, the spatial demand of the cages limits the density of the closely packed surface structure, and the density should remain the same even if the carboxylate density is increased. In Figure 4d, the measured SPR response after bowl injection on MHDA SAMs is plotted alongside the theoretically calculated coverages to compare the theoretical density based on available anchoring sites against the increase in SPR response. The two curves were scaled to achieve similar heights in the region approaching saturation. While the experimental SPR curve shows a smoother transition than the theoretical prediction, the saturation occurs at a similar surface density of carboxylate groups of ≈2.5%. The smoother shape most likely originates from a lower packing order and potentially carboxylate groups that are too close together or in an unfavorable position on the surface, reducing the overall binding ability.

AFM measurements were performed in ethanol to compare the surface morphology of a pristine SAM and a SAM after the immobilization of the coordination cages with encapsulated C_60_ (Figure 5). For the experiments, mixed SAMs prepared in a 1% MDDA solution were used. The AFM images of the gold facet show distinct regions of varying height, revealing that the 1% MDDA monolayers have defects in the coverage. After incubation in the guest-encapsulated coordination cage solution (C_60_@bowl), the SAM roughens and small features extend from the monolayer surface. Line scans (Figure 5B) revealed a height of ∼1 nm for the circular structures, a size comparable to the size of C_60_ which is in the order of 0.7 nm, and reasonable for the expected size increase by encapsulation in the slightly larger bowl.^32^ AFM images revealed an RMS value of 0.24 ± 0.04 nm for the 1% MDDA SAM, which increased to 0.26 ± 0.05 nm upon incubation in the C_60_@bowl solution.

**Figure 5:**
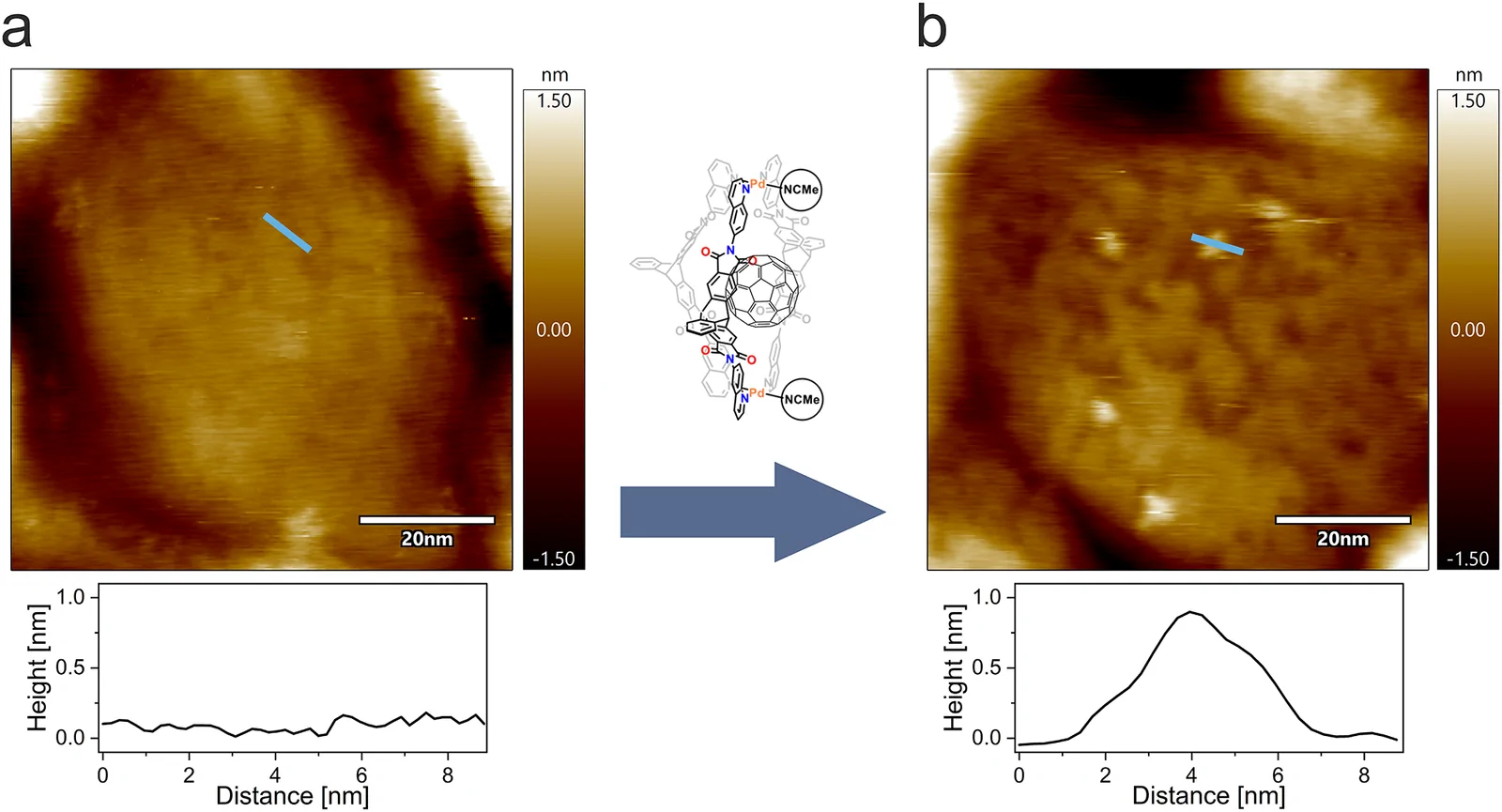
AFM analysis of the surface binding of bowls that contain a C_60_ guest. A 75 × 75 nm topographic image of a mixed SAM surface measured in ethanol, prepared with a solution of 1% carboxyl-terminated MDDA and 99% methyl-terminated DDT on Au. (a) Before and (b) after incubation in a guest-encapsulated coordination cage solution.

By SPR spectroscopy, the surface immobilization process of the bowl on the MDDA SAM surface was compared with that of the guest-encapsulated C_60_@bowl complex (Figure 6a). The sensorgram showed similar trends during solution injection, differing only in the maximum SPR response reached. The guest-encapsulated C_60_@bowl showed a postdissociation response of 2657 ΔμRIU, whereas the empty bowl showed a response of 1050 ΔμRIU (Figure 6b). In correlation with the higher response, the association rate of the guest-encapsulated C_60_@bowl was higher at 15.9 M^–1^s^–1^ than that of the empty bowl, which had an association rate of 13.7 M^–1^s^–1^ (Figure 6c).

**Figure 6:**
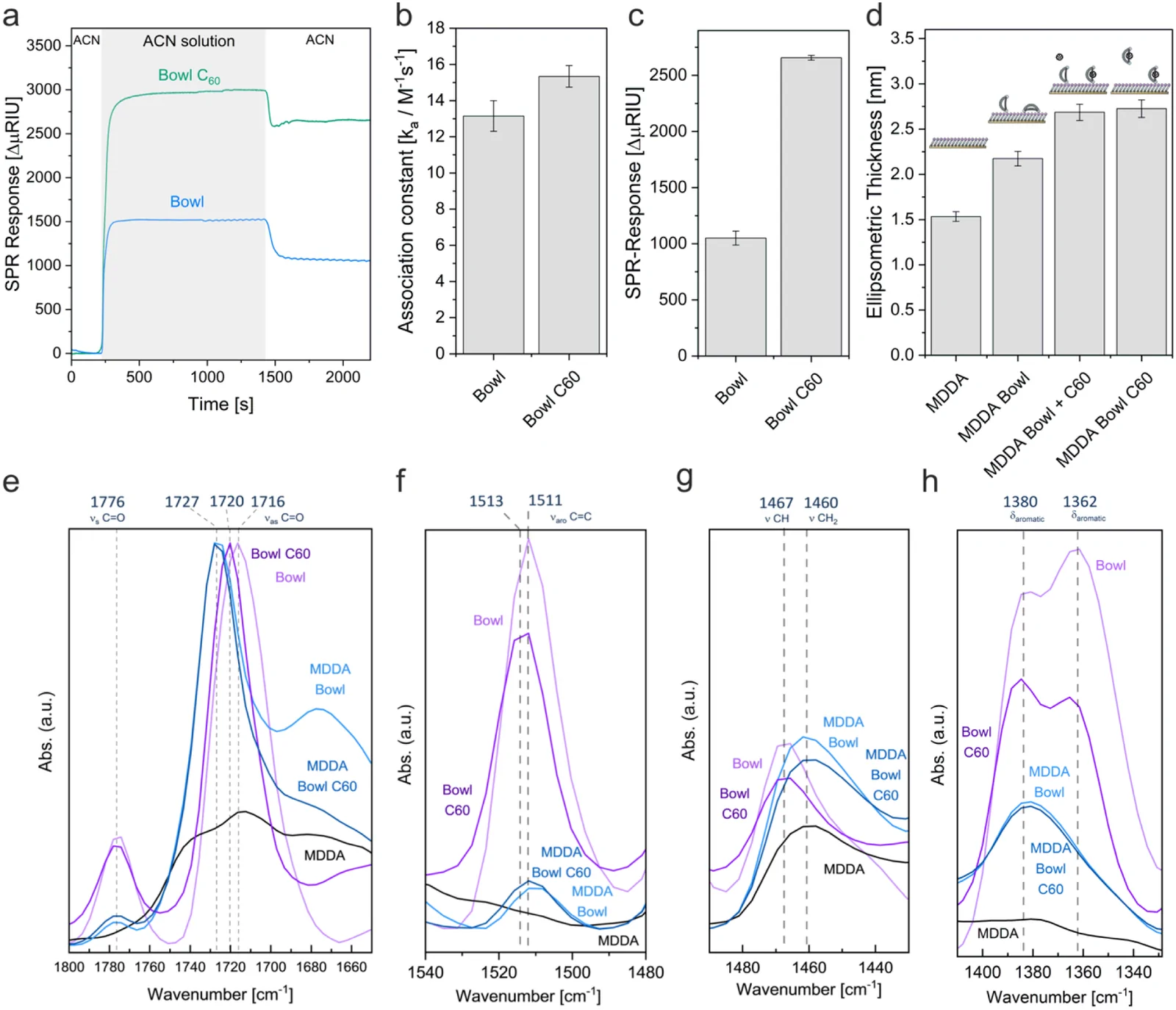
Analysis of the C_60_ interaction with SurSCCs using SPR, spectroscopic ellipsometry, and ATR-FTIR (a) Representative SPR sensorgram of the injection of 0.35 mM bowl solution and 0.35 mM bowl with encapsulated C_60_ (C_60_@bowl) in acetonitrile on an 100% MDDA-functionalized SPR chip. Bowl solutions were injected for 20 min at a flow rate of 10 μL/min. (b) SPR response (ΔμRIU) of MDDA after injection of 0.35 mM bowl solutions in acetonitrile. Samples were injected for 20 min with a flow rate of 10 μL/min. Values obtained after dissociation at *t* = 2000 s. Values are the mean of at least three individual measurements. The error bars represent the standard deviation. (c) Corresponding association constants (*k*_a_) were obtained from fitting of the experimental data. (d) Thickness of the SAM surface before and after incubation in solutions of the different adsorbents. Values are the mean of at least three individual measurements. The error bars represent the standard deviation. (e–h) ATR-FTIR spectra of the bowl-shaped coordination cage and the guest-encapsulated cage, the SAM surface MDDA, and the surface-absorbed coordination cages. Selected spectral regions are shown as insets: (e) 1800 to 1650 cm^–1^, (f) 1540 to 1480 cm^–1^, (g) 1490 to 1430 cm^–1^, (h) 1420 to 1330 cm^–1^.

Spectroscopic ellipsometry was used to determine the increase in thickness after incubation in the various sample solutions. A comparison was made to determine whether a distinction could be made between the guest uptake before immobilization (C_60_@bowl) and the guest uptake of the already-immobilized bowl (bowl + C_60_) (Figure 6d). After bowl immobilization, the thickness increased by 0.64 to 2.4 nm. After incubation of the immobilized bowl in a C_60_ solution, the thickness increased to 2.8 nm, whereas immobilization of the guest-encapsulated bowl resulted in a comparable thickness of 2.7 nm.

By ATR-FTIR spectroscopy, the guest encapsulation was analyzed in greater detail. Therefore, the spectra of the bowl and MDDA bowl were compared to the bowl with incorporated C_60_ (C_60_@bowl) and the surface-immobilized bowl with incorporated C_60_ (MDDA C_60_@bowl) (Figure 6d–h). The pure bowl and the bowl C_60_ material showed almost the same peaks, but with small shifts in the wavenumbers of the different vibrational modes. The peak of the antisymmetric C=O vibration at 1716 cm^–1^ shifted to 1720 cm^–1^, and the peak assigned to the aromatic C=C vibration at 1511 cm^–1^ shifted to 1513 cm^–1^. The peaks assigned to the deformation vibrations of the aromatic ring systems at 1380 cm^–1^ and 1362 cm^–1^ shifted to higher wavenumbers, 1384 cm^–1^ and 1365 cm^–1^. The observed changes due to the binding of the C_60_ could be due to π-π stacking with the aromatic functions of the cage system, altering the local electron densities. For the surface-immobilized bowls, the spectral differences upon guest encapsulation were more subtle. The peak at 1673 cm^–1^ for MDDA bowl, already assigned above to the C = O vibration of the system, is less intense and not as pronounced for C_60_@bowl. Additionally, the peak at 1511 cm^–1^ for MDDA bowl, assigned to the aromatic C=C vibrations, shifted to slightly higher wavenumbers upon the introduction of the guest’s π-system.

To analyze the directed assembly of bowl-shaped coordination cages on carboxy-terminated SAMs, different analytical methods were combined. In situ flow experiments using SPR spectroscopy revealed the absorption of the cages and their building blocks from solution. Individual injections led to noticeably higher SPR responses of 1050 ΔμRIU for the cage immobilized on MDDA, compared with 628 ΔμRIU for the Pd salt and 159 ΔμRIU for the ligand. Spectroscopic ellipsometry measurements support this trend and show a stronger thickness for the adsorbed cage (0.64 nm) than for the adsorbed Pd salt (0.21 nm) or the ligand (0.11 nm). These differences in absorption suggest that the entire cages bind to the surface. The kinetic evaluation of the association rates supports this conclusion as the cages have almost twice as high association rate (*k*_a_ = 13.7 M^–1^s^–1^), suggesting a higher binding affinity to the surfaces than its building blocks. Comparison of the IR spectra of the coordination cage and the ligand showed several specific shifts, which can be attributed to the coordination of the ligand to the Pd­(II) metal center of the cages. After immobilization of the cages and the ligand on the SAM surface, the IR spectra revealed the same peak shifts as for the free cage and ligand, further supporting that the cages remain intact after surface immobilization. The water contact angle (WCA) data support the conclusions from the analytical data and show a clear difference after absorption of the cage and the individual components, as the surfaces with immobilized cages became more hydrophobic (61°) than the surfaces after incubation in the Pd salt solution (42°) or the ligand solution (37°). The combination of the different surface analytical data provides strong evidence that the cages are immobilized intact on the surface.

If cage immobilization occurs specifically via the carboxy groups of the SAM as binding sites, then the amount of bound bowl-shaped cages would be expected to increase in proportion to the number of binding sites. However, due to the size of the cages and their steric demand on the SAM surfaces, this increase should level off if a certain number of binding sites is exceeded since not all binding sites are accessible. To show this experimentally, mixed SAMs were prepared by combining the carboxy-terminated SAM with the alkyl-terminated DDT to systematically adjust the number of binding sites. As theoretically expected, the measured SPR responses show a linear increase at low carboxylate contents in the mixed SAMs, which saturate at higher carboxylate contents when a high surface coverage of the bowls is achieved. This indicates that the cage binding is specific and can be controlled by a variation of the number of carboxylates present on the SAM surface. When assembling a mixed SAM from MDDA with DDT, the carboxy function of the MDDA should not stick out of the final SAM as DDT and MDDA have the same alkyl chain length (C_11_). The use of a carboxy-terminated SAM (MHDA) with a longer alkyl chain (C_15_) should allow the carboxy group to slightly protrude from the interface. A comparison of the SPR response with increasing carboxy content for MDDA and MHDA shows similar maximum SPR response values, suggesting that the availability of carboxy functions is similarly high for both chain lengths.

To investigate the influence of the presence of C_60_ incorporated into the cages as a guest on the surface affinity of the cages, in situ flow experiments were performed using SPR spectroscopy. Therefore, the bowl-shaped coordination cages with C_60_ present as a guest were injected, revealing an SPR response of 2657 ΔμRIU for the guest-encapsulated cages that was higher than the one of the empty cages of 1050 ΔμRIU. In correlation with the SPR response, spectroscopic ellipsometry revealed a stronger increase in thickness after absorption of the guest-encapsulated cage (1.19 nm) than of the empty one (0.64 nm). The higher thicknesses most likely reflect the increased refractive index of the coordination cages with the guest being present but might also contain an enhanced coverage on the surface. Comparison of the IR spectra of the guest-encapsulated cage and the empty cage in bulk state showed small shifts to higher wavenumbers, indicating the presence of the π-system of the C_60_ in the bowls, which is very likely, as C_60_ is insoluble in the used acetonitrile and thus any removal is very unlikely.

While the probability to successfully obtain topographical images of the bowl-shaped coordination cage on SAM surfaces is very low due to their soft organic structures, AFM images of C_60_ have already been successfully recorded.^33,34^ For this reason, AFM images of the cage with encapsulated C_60_ were acquired to use C_60_ as a marker and provide the system with the necessary stability. Mixed SAM surfaces with 1% MDDA were produced to minimize the number of binding sites. This was intended to ensure that the bowl C_60_ structures were as isolated as possible and to enable individual topographic images to be acquired. The AFM images revealed structures with a height of approximately 1 nm. This height is slightly greater than the C_60_ diagonal of 0.7 nm^32^ and can therefore be attributed to the C_60_@bowl structures. The surface roughness (RMS) of the SAM surface increased from 0.24 to 0.26 nm upon immobilization of the C_60_@bowl structures. The AFM images also show that bowls attach rather individually, with a low tendency to form aggregates on the surface. Spectroscopic ellipsometry reveals that the thickness increase of surface-bound cages after C_60_ exposure yields similar thickness increases as the assembly of C_60_-bound bowls, which shows the general possibility for in situ adsorption studies in immobilized bowls at interfaces and suggests the high potential of SurSCCs for host–guest studies.

## Conclusion

Bowl-shaped coordination cages were immobilized on carboxy-terminated SAMs. The analysis of the absorption of the cages by SPR spectroscopy, spectroscopic ellipsometry, and ATR-FTIR spectroscopy revealed higher absorption of the bowl-shaped coordination cages compared to its individual building blocks, indicating a higher affinity for the intact bowl-shaped cages that offer Pd­(II) coordination sites to bind to the surface-mounted carboxylates. The specificity of selective binding to the carboxylic acid groups was proven and enabled the controlled variation of the number of binding sites on the SAM surface and, consequently, the density of immobilized cages. Upon incorporation of the guest C_60_, the binding affinity of the cages was increased, as verified by SPR spectroscopy and spectroscopic ellipsometry. The binding of the C_60_-containing cages was additionally shown by AFM, in which topographic images showed structures that match the expected size of the C_60_-containing bowls. Our results highlight the possibility of the directed immobilization of coordination cages on surfaces and show how the surface density can be controlled by the number of available binding sites. Our approach demonstrates a controlled method for a directed immobilization of SurSCCs on surfaces with tremendous potential for spectroscopic studies of individual coordination cages and their host–guest interactions. In light of the current activities of many research groups to develop new supramolecular cage types with selectivities for a wide range of guests such as various natural products, chiral pharmaceuticals, toxins, and valuable chemical resources,^3,35^ our herein reported methodology and proof-of-principle results enable the advancement of new sensor technologies and applications in fields such as medical diagnostics and environmental analytics.

## References

[1] Cook T. R., Zheng Y. R., Stang P. J. (2013). Metal-Organic
Frameworks and Self-Assembled
Supramolecular Coordination Complexes: Comparing and Contrasting the
Design, Synthesis, and Functionality of Metal-Organic Materials. Chem. Rev..

[2] Pullen S., Tessarolo J., Clever G. H. (2021). Increasing Structural and Functional
Complexity in Self-Assembled Coordination Cages. Chem. Sci..

[3] Cox C. J. T., Hale J., Molinska P., Lewis J. E. M. (2024). Supramolecular
and Molecular Capsules, Cages and Containers. Chem. Soc. Rev..

[4] Ham R., Nielsen C. J., Pullen S., Reek J. N. H. (2023). Supramolecular
Coordination Cages for Artificial Photosynthesis and Synthetic Photocatalysis. Chem. Rev..

[5] Piskorz T. K., Martí-Centelles V., Spicer R. L., Duarte F., Lusby P. J. (2023). Picking the Lock
of Coordination Cage Catalysis. Chem. Sci..

[6] Chen S., Chen L. J. (2022). Metal–Organic
Cages: Applications in Organic
Reactions. Chemistry.

[7] Frank M., Funke S., Wackerbarth H., Clever G. H. (2014). SERS Spectroscopic
Evidence for the Integrity of Surface-Deposited Self-Assembled Coordination
Cages. Phys. Chem. Chem. Phys..

[8] Menozzi E., Pinalli R., Speets E. A., Ravoo B. J., Dalcanale E., Reinhoudt D. N. (2004). Surface-Confined
Single Molecules: Assembly and Disassembly
of Nanosize Coordination Cages on Gold (111). Chem. – Eur. J..

[9] Kudernac T., Lei S., Elemans J. A. A. W., De Feyter S. (2009). Two-Dimensional
Supramolecular Self-Assembly: Nanoporous Networks on Surfaces. Chem. Soc. Rev..

[10] Ryan H. P., Haynes C. J. E., Smith A., Grommet A. B., Nitschke J. R. (2021). Guest Encapsulation
within Surface-Adsorbed Self-Assembled Cages. Adv. Mater..

[11] Ludden M. J. W., Reinhoudt D. N., Huskens J. (2006). Molecular Printboards: Versatile
Platforms for the Creation and Positioning of Supramolecular Assemblies
and Materials. Chem. Soc. Rev..

[12] Troshin P. A., Hoppe H., Peregudov A. S., Egginger M., Shokhovets S., Gobsch G., Sariciftci N. S., Razumov V. F. (2011). [70] Fullerene-Based
Materials for Organic Solar Cells. ChemSusChem.

[13] Prato M. (1997). 60 Fullerene
Chemistry For Materials Science Applications. J. Mater. Chem..

[14] García-Simón C., Costas M., Ribas X. (2016). Metallosupramolecular Receptors for
Fullerene Binding and Release. Chem. Soc. Rev..

[15] Chen B., Holstein J. J., Horiuchi S., Hiller W. G., Clever G. H. (2019). Pd­(II)
Coordination Sphere Engineering: Pyridine Cages, Quinoline Bowls,
and Heteroleptic Pills Binding One or Two Fullerenes. J. Am. Chem. Soc..

[16] Chang X., Xu Y., von Delius M. (2024). Recent Advances
in Supramolecular Fullerene Chemistry. Chem.
Soc. Rev..

[17] Martin, N. ; Nierengarten, J. Supramolecular Chemistry of Fullerenes and Carbon Nanotubes; Wiley, **2012**. 10.1002/9783527650125

[18] Zhang D., Ronson T. K., Nitschke J. R. (2018). Functional
Capsules via Subcomponent
Self-Assembly. Acc. Chem. Res..

[19] Hirsch, A. ; Brettreich, M. Fullerenes: : Chemistry and reactions; John Wiley & Sons, **2006**.

[20] Effing J., Jonas U., Jullien L., Plesnivy T., Ringsdorf H., Diederich F., Thilgen C., Weinstein D. (1992). C 60 and C
70 in a Basket? – Investigations of Mono- and Multilayers from
Azacrown Compounds and Fullerenes. Angew. Chem.
Int. Ed. Engl..

[21] Andersson T., Nilsson K., Sundahl M., Westman G., Wennerström O. (1992). C_60_ Embedded in γ-Cyclodextrin:
A Water-Soluble Fullerene. J. Chem. Soc., Chem.
Commun..

[22] Matsumoto K., Kusaba S., Tanaka Y., Sei Y., Akita M., Aritani K., Haga M., Yoshizawa M. (2019). A Peanut-Shaped
Polyaromatic Capsule: Solvent-Dependent Transformation and Electronic
Properties of a Non-Contacted Fullerene Dimer. Angew. Chem. Int. Ed..

[23] Ronson T. K., League A. B., Gagliardi L., Cramer C. J., Nitschke J. R. (2014). Pyrene-Edged
Fe II 4 L 6 Cages Adaptively Reconfigure During Guest Binding. J. Am. Chem. Soc..

[24] Hasegawa S., Meichsner S. L., Holstein J. J., Baksi A., Kasanmascheff M., Clever G. H. (2021). Long-Lived C60Radical Anion Stabilized Inside an Electron-Deficient
Coordination Cage. J. Am. Chem. Soc..

[25] Dong J., Yin C., Zhang Z., Wang X., Li H., Zhang Q. (2014). Hydrogen-Bonding
Interactions and Molecular Packing in Polyimide Fibers Containing
Benzimidazole Units. Macromol. Mater. Eng..

[26] Bienz, S. ; Bigler, L. ; Fox, T. ; Hesse, M. ; Meier, H. Spektroskopische Methoden in Der Organischen Chemie; 9, ed.; Thieme, **2016**.

[27] Stamboliyska B. A., Binev Y. I., Radomirska V. B., Tsenov J. A., Juchnovski I. N. (2000). IR Spectra
and Structure of 2,5-Pyrrolidinedione (Succinimide) and of Its Nitranion:
Experimental and Ab Initio MO Studies. J. Mol.
Struct..

[28] Özel A. E., Büyükmurat Y., Akyüz S. (2001). Infrared-Spectra
and Normal-Coordinate Analysis of Quinoline and Quinoline Complexes. J. Mol. Struct..

[29] Arnold R., Azzam W., Terfort A., Wöll C. (2002). Preparation,
Modification, and Crystallinity of Aliphatic and Aromatic Carboxylic
Acid Terminated Self-Assembled Monolayers. Langmuir.

[30] Folkers J.
P., Laibinis P. E., Whitesides G. M. (1992). Self-Assembled Monolayers of Alkanethiols
on Gold: Comparisons of Monolayers Containing Mixtures of Short- and
Long-Chain Constituents with Methyl and Hydroxymethyl Terminal Groups. Langmuir.

[31] Ulman A., Eilers J. E., Tillman N. (1989). Packing and
Molecular Orientation
of Alkanethiol Monolayers on Gold Surfaces. Langmuir.

[32] Kroto H. W., Heath J. R., O’Brien S. C., Curl R. F., Smalley R. E. (1985). C60: Buckminsterfullerene. Nature.

[33] Zhang P., Lu J., Xue Q., Liu W. (2001). Microfrictional Behavior of C60 Particles
in Different C60 LB Films Studied by AFM/FFM. Langmuir.

[34] Pawlak R., Kawai S., Fremy S., Glatzel T., Meyer E. (2011). Atomic-Scale
Mechanical Properties of Orientated C60 Molecules Revealed by Noncontact
Atomic Force Microscopy. ACS Nano.

[35] Wang Y.-P., Zhang Y., Duan X.-H., Mao J.-J., Pan M., Shen J., Su C.-Y. (2024). Recent
Progress in Metal-Organic
Cages for Biomedical Application: Highlighted Research during 2018–2023. Coord. Chem. Rev..

